# Reverse Transcriptase and Cellular Factors: Regulators of HIV-1 Reverse Transcription

**DOI:** 10.3390/v1030873

**Published:** 2009-11-10

**Authors:** Kylie Warren, David Warrilow, Luke Meredith, David Harrich

**Affiliations:** 1 Division of Infectious Diseases, Queensland Institute of Medical Research, Brisbane, QLD, Australia; E-Mails: kylieW@qimr.edu.au (K.W.); davidW@qimr.edu.au (D.W.); lukeMe@qimr.edu.au (L.M.); 2 School of Natural Sciences, University of Western Sydney, Hawkesbury, NSW, Australia; 3 Griffith Medical Research College, a joint program of Griffith University and the Queensland Institute of Medical Research, QIMR, Herston, QLD, 4006, Australia

**Keywords:** HIV-1, reverse transcriptase, HuR, AKAP149, DNA topoisomerase I, Sin3a, HDAC1, SAP18, SAP, SMN, Gemin2, lysl-tRNA synthetase, INI1, DHX9, APOBEC3G

## Abstract

There is ample evidence that synthesis of HIV-1 proviral DNA from the viral RNA genome during reverse transcription requires host factors. However, only a few cellular proteins have been described in detail that affect reverse transcription and interact with reverse transcriptase (RT). HIV-1 integrase is an RT binding protein and a number of IN-binding proteins including INI1, components of the Sin3a complex, and Gemin2 affect reverse transcription. In addition, recent studies implicate the cellular proteins HuR, AKAP149, and DNA topoisomerase I in reverse transcription through an interaction with RT. In this review we will consider interactions of reverse transcription complex with viral and cellular factors and how they affect the reverse transcription process.

## 1. Overview

HIV-1 is a member of the lentivirus genus of the family *Retroviridae*. Like all retroviruses, HIV-1 is a positive sense (+) single stranded (ss) RNA virus that must integrate its genetic material into the host DNA chromosomes to complete its life cycle. This is accomplished in part by the complicated process of reverse transcription in which the retroviral enzyme reverse transcriptase (RT) copies the (+)ssRNA into double strand DNA. HIV-1 initially enters a target cell as a permeable protein shell composed of viral capsid and other proteins and protects the initial steps of reverse transcription from cellular detection and degradative processes. A number of different studies now point to interactions between the reverse transcription complex, which minimally includes RT and integrase (IN), with cellular proteins enabling the production of full length proviral DNA. Here we review the viral components of reverse transcription and subsequently critically consider recently described cellular factors that help or hinder HIV-1 reverse transcription by directly or indirectly affecting RT.

## 2. RT Structure and Function

**Figure 1.**
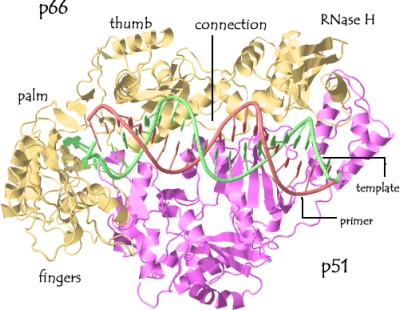
RT Structure. The p66 subunit is shown in yellow and p51 subunit in purple. A vRNA:tRNA structure is juxtaposed on the RT molecule, where the vRNA is green and the tRNA is brown. The model was generated and adapted using RasMol and the PBD file ID# 1R0A [2].

RT is an asymmetric heterodimeric enzyme consisting of a 560-amino-acid 66-kDa subunit (p66) and a 440-amino-acid 51-kDa subunit (p51) [1] (Figure 1). The heterodimer contains a single active DNA polymerisation site, an RNase H active site and an RNA binding site.

The p66 subunit of RT can be divided structurally into the polymerase and RNase H domains. The polymerase domain can be further differentiated into subdomains that have been described as structurally resembling a right hand, forming the fingers, palm, thumb, and connection subdomains [1,3–6]. In the heterodimer, p66 has an “open” conformation, which facilitates binding to the incoming RNA template/tRNA complex during reverse transcription [1,4,7].

While p51 has the same polymerase subdomains as p66, the relative orientations of the individual subdomains differ markedly. p51 lacks the cleft domain, as the active polymerase site is buried within the structure [4]. Subsequently p51 forms a “closed” conformation and plays a largely structural role to stabilize the RT heterodimer [8,9], although there is some evidence that the p51 subunit may be required for correct binding of the tRNA in the RT binding cleft [10].

## 3. Formation of the HIV-1 Reverse Transcription Initiation Complex in the Mature Virus Particle

Interactions between RT and cellular factors are possible during early and late phases of virus replication. Two viral proteins, 55 kDa Gag and 160 kDa Gag-Pol, and two unspliced copies of viral genomic mRNA form the bulk of nascent virions assembled at the cellular plasma membrane. After budding, the virion precursor proteins are processed by the viral protease (PR) into matrix protein (MA), capsid protein (CA) and nucleocapsid protein (NC) and p6, and two small spacer proteins from Gag; and three viral enzymes: PR, reverse transcriptase (RT) and integrase (IN) from the Pol component of Gag-Pol. CA self-assembles into a fullerene cone [11–13] which contains the genomic viral RNA (vRNA) associated with NC, RT, IN, MA and Vpr. Within this CA cone a prototypical reverse transcription complex (RTC) is established in the mature virion [reviewed in 14]. At least two cellular factors have been linked to formation of the RTC. A cellular tRNA primer is packaged in particles by interaction with Gag with the aminoacyl-tRNA^Lys,3^ synthetase (LysRS) which has tRNA^Lys,3^ bound [15]. Additional specificity is achieved as only “charged” LysRS molecules (those with tRNA^Lys,3^ bound) are recruited by additional interaction with the thumb and connection domains of Pol (in the Gag-Pol precursor) [16]. The annealing of tRNA^Lys,3^ requires the RNA chaperone activity of NC [17,18]. Additionally, the mechanism by which the tRNA primer is bound to the viral RNA involves a complicated mechanism involving interactions between tRNA^Lys,3^, LysRS and RT, in which contact between Gag, Gag-Pol and LysRS facilitate annealing of non-actylated tRNA^Lys,3^ to the PBS [16]. RT binding to LysRS is mediated primarily by the thumb domain, although analysis of truncated RT proteins suggested that multiple domains of RT are important.

**Figure 2.**
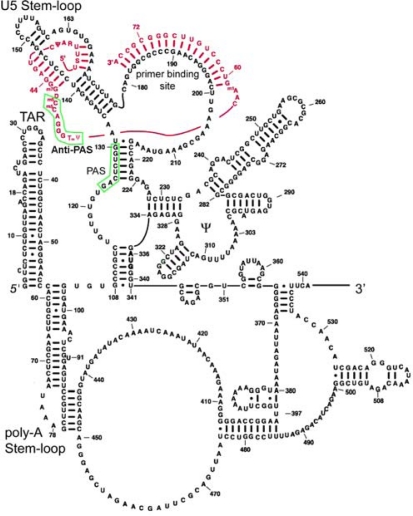
Model of the HIV-1 RNA structure from +1 to +540. Proposed interactions between tRNA^Lys,3^ and the U5 Stem-loop are indicated. The viral RNA sequence is colored black and the tRNA^Lys,3^ sequence is in red. TAR: transactivation response element. Poly-A Stem loop contains the poly-adenylation signal AAUAAA functions on the 3′ long terminal repeat. The ψ indicates the vRNA packaging signal. The PAS on the vRNA and the anti-PAS on tRNA^Lys,3^ are boxed in green. Adapted from Wilkinson *et al.* (2008) [24].

RNA interactions between vRNA and tRNA form an RNA architecture which is required for initiation of reverse transcription (Figure 2). Although some structural features are controversial, an RNA structure determined by chemical and enzymatic probing of synthetic or viral HIV-1 RNA annealed to tRNA^Lys,3^ [19–23], and recently using high-throughput SHAPE (selective 2′-hydroxyl acylation analysed by primer extension) in intact viruses, with *ex vivo* virus RNA or synthetic RNA [24,25] are similar. This complex is thought to form an RNA “scaffold” bound by reverse transcriptase, nucleocapsid and possibly other viral and cellular proteins, which as a whole form the reverse transcription initiation complex [20,21]. A region referred to as the primer activation signal (PAS), located approximately 50 nucleotides upstream of the PBS, enhances the usage of tRNA^Lys,3^ and appears to regulate the initiation reaction [26–28]. However, an interaction between tRNA and PAS was not identified by SHAPE analysis suggesting that it may be transient. Generally, mutation of these RNA regions often negatively affects the initiation of reverse transcription. Finally, RT has contact sites in the vRNA:tRNA complex in addition to the known contact made by p66 in the substrate cleft where the primer:template are clamped [29]. The p66 fingers subdomain make contacts to the vRNA:tRNA complex most likely in U5 just ahead of the tRNA primer.

*In vitro* kinetics studies of reverse transcription indicate that the initiation reaction is significantly slower than elongation of the nascent DNA strand by RT. The initiation phase incorporates 1 to 5 nucleotides to the tRNA primer, followed by a more rapid elongation phase where the polymerization rates increase about 3000-fold [30,31]. Curiously, typical viral genomic RNAs are unextended or have a di-nucleotide extension on the primer prior to initiation of reverse transcription [32]. The initiation step of HIV-1 reverse transcription is inhibited by a poorly defined mechanism possibly involving the RNA structure described, by low nucleotide concentrations or perhaps by an unknown factor [28,33,34].

## 4. The Complexities of Integrase and Reverse Transcriptase

IN is a 288 amino acid, 31 kDa protein making up the C-terminal end of the Gag-Pol protein that has three domains consisting of the N-terminal (residues 1–50), catalytic core (residues 51–212), and C-terminal domain (CTD, residues 213–288). IN is active as a dimer with its primary function being to integrate the viral DNA product [35]. Its penultimate role is to prepare the viral DNA for insertion into the host chromosome. To accomplish this task, IN remains associated with the reverse transcription complex, as the double strand viral DNA is made, and transitions into a pre-integration complex (PIC) capable of ligating proviral DNA into a chromosome [36]. IN can directly bind to RT [37–40] and deletion analysis of IN indicates that the CTD, which is a non-specific DNA binding domain [41–44], is necessary and sufficient to bind RT [39,40]. Conversely, RT reportedly has two IN-binding sites; one each in the finger-palm and connection subdomain regions [39]. The association between IN and the RTC appears to be obligatory, as some IN mutants negatively affect proviral DNA synthesis [37,45,46]. Mutations that inhibit reverse transcription are referred to as type II, whereas type I mutations affect integration [47,48]. Recently nuclear magnetic and surface plasmon resonance (NMR and SPR, respectively) experiments using a CTD-derived peptide revealed a putative binding surface on IN [49]. One mutation on this surface, K258A, substantially reduced CTD and RT binding. An unanswered question about CTD and other type II mutants is whether they act directly on RT, or if an interaction between IN and other viral or cellular factors are involved. Evidence that IN and RT interaction is required after infection has been inferred from genetic experiments, but rigorous correlation between IN and RT interaction and defective reverse transcription requires confirmation.

Initiation of reverse transcription refers to the addition of the first five deoxynucleotides to the tRNA^Lys,3^ primer. *In vitro* experiments using recombinant IN and RT indicate that IN can increase the efficiency of initiation [50]. Dobard *et al.* showed that IN-mediated initiation also requires natural tRNA^Lys,3^ suggesting that the stability of the RT:primer/template complex is enhanced when IN is present. However IN also increases RT elongation dramatically. One possible explanation is that IN binding to RT increases the affinity of RT for the template RNA strand, as evident in competition assays using molecular traps that can bind RT that disengages the RNA template during DNA synthesis. It is unclear why IN is unable to enhance RT elongation when short RNA oligonucleotides are used to prime reverse transcription. It is possible that tRNA^Lys,3^ is required to form an initial RT-IN complex, which is maintained during extended DNA synthesis. Another possibility is that tRNA^Lys,3^ facilitates exposure of an IN binding site on RT. However, this is at odds with the observation that the RT-IN interaction does not require an RNA co-factor [37].

## 5. IN-Binding Cellular Factors that Affect Reverse Transcription

Yeast-two hybrid systems used to screen libraries of human genes have identified cellular factors that interact with integrase [51–55], which were subsequently confirmed by *in vitro* protein GST pull-down and co-immunoprecipitation assays where IN was over expressed in cell culture. These include the cellular proteins integrase interactor 1 (INI1, hSNF5) [53,54], sin3A-associated protein (SAP18), histone deactylase 1 (HDAC1) [52] and survival motor neuron (SMN)-interacting protein 2 (Gemin2) [55]. Given that mutation of IN can affect RT activity, it is not surprising that some IN binding cellular proteins also affect reverse transcription. Some of these cell proteins and their effects are described in the section below.

### 5.1. A role for INI1 in early events during HIV-1 replication?

INI1 is a 385 amino acid, 44 kDa nuclear protein (also called hSNF5, BAF47 and SMARCB1) component of the SWI/SNF chromatin remodeling complex that is involved in tumor suppression [56]. INI1 binds to HIV-1 IN as well as proteins from other viruses including E1 from human papillomavirus 18 and the EBNA-2 from Epstein-Barr virus regulating virus replication [57,58]. Structurally, INI1 has two imperfect amino acid repeat regions, RPT1 and RPT2, and a coiled coil domain that is conserved in other related mammalian and yeast proteins, termed the homology region 3 (HR3). INI1 is capable of multimerization and forms high molecular weight complexes primarily requiring both RPT motifs (the N-terminal domain also appears to play a role) [59]. Whereas only RPT1 is required to bind IN [53], a region adjacent to RPT1 in INI1 is required to bind DNA along the minor groove. Although primarily a nuclear protein, INI1 has a “masked” nuclear export signal (NES) within RPT2 that is critical for its ascribed cellular functions [60].

INI1 affects HIV-1 replication at several points in the HIV-1 life cycle. In line with its role in the SWI/SNF chromatin remodeling complex, INI1 can affect both basal and Tat mediated HIV-1 gene expression [61,62]. INI1 is also required for particle production as exogenously expressed INI1 can partially rescue reduced HIV-1 production in cells lacking INI1 [63]. INI1 is specifically packaged in HIV-1, but not other retroviruses, and is regulated by a direct interaction between INI1 and the IN domain of the HIV-1 Gag-Pol protein [64]. Several lines of evidence suggest INI1 may be required during early steps of HIV-1 replication. Immediately following infection by HIV-1, INI1 in a target cell is rapidly and transiently exported from the nucleus by an hCRM1/exportin1 nuclear export pathway [60,65]. INI1 associates with the incoming RTC/PIC in the cytoplasm [65], and can stimulate IN activity *in vitro* [53]. Thus, INI1 is a candidate cellular factor regulating RTC/PIC activity (Table 1).

**Figure 3.**
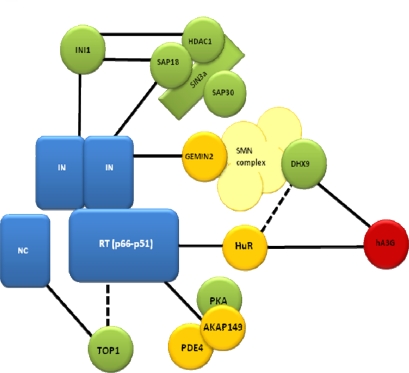
Schematic of RTC interacting cellular factors. Known direct (—) and indirect (---) associations between RTC (blue) and host factors that affect reverse transcription. Cellular factors are incorporated into virion particles (green), not incorporated (yellow) or conditionally incorporated (red).

Three recent reports investigating early HIV-1 replication using different experimental approaches reached different conclusions regarding INI1 function during early replication. Sorin *et al.* used two rhabdoid tumor-derived cell lines, MON and STA-WTI, which are devoid of functional INI1, to produce HIV-1-like particles [63]. MON and STA-WTI cells transfected with an HIV-1 derived lentiviral plasmid made >10-fold less virus than 293T cells. Virus production could be increased by exogenously expressing INI1. Interestingly, the INI1 negatively regulated HIV-1 produced from MON cells, but not STA-WTI cells, exhibited a sharp defect in reverse transcription and virus infectivity. Exogenously expressed INI1 in MON cells did not complement these defects indicating that an additional unknown factor was involved in early replication. Curiously, exogenous RT activity in MON produced virions was sharply reduced. This is unexpected as RT levels in INI1+ and INI1-virions are similar; suggesting that a potent RT inhibitor made in MON cells may be packaged in virions. These experiments affirm INI1 as an important factor for virus particle production, but suggest that another factor perhaps acting in concert with INI1 is a regulator of reverse transcription (see Sin3a complex below) (Figure 3).

Ariumi *et al.* investigated INI1 and its role in Tat transactivation of HIV-1 gene expression [61]. Here, INI1 was stably downregulated in CD4+ HeLa-derived P4.2 cells and infected with an HIV-1-derived lentiviral vector expressing GFP using a CMV promoter (to bypass a need for Tat to monitor gene expression). In this study, no change in virus infectivity was observed. Hence, INI1 had no affect on events from fusion to integration, suggesting that INI1 was not essential for RTC or PIC function.

Maroun *et al.* down-regulated INI1 in Jurkat or P4.2 cells transiently using siRNA [66]. In this case, HIV-1 replication was enhanced in both cell types indicating that INI1 negatively affected HIV-1 replication. Infection of INI1 depleted cells resulted in increased levels of 2-LTR circular DNA and integrated provirus suggesting that interaction between INI1 and IN somehow inhibited integration. A specific IN mutant protein, K71R, blocked the INI1:IN interaction, and HIV-1 with this point mutation replicated better leading to the conclusion that INI1:IN complex was detrimental for HIV-1 replication. A similar conclusion was previously reported for the homologous recombination repair protein RAD52 [67].

It is clear that INI1 has diverse roles in HIV-1 replication including virus gene expression and virus assembly and integration. Sometimes, virus made by cells lacking INI1 display an RT defect, but this could be attributed to factors other than INI1. Discrepancies in INI1 function between these 3 studies may be due to differences in INI1 expression or cell type specific effects. The suggestion that INI1 has innate antiviral activity is intriguing, however further research is required to clarify the role INI1 in cells following HIV-1 infection.

### 5.2. Sin3a-HDAC1complex and early events in HIV-1 replication

Recently, several Sin3a-HDAC1 complex proteins were found to be specifically packaged in HIV virions [52] (Table 1)(Figure 3). In cells, the Sin3a complex is involved in chromatin remodeling leading to transcriptional repression [see 68]. At the heart of this complex is Sin3, a scaffold protein able to interact with several proteins simultaneously, which facilitates interaction between numerous proteins including transcription factors, histone modifying proteins, and various Sin3a associated protein (SAP) including SAP18 and SAP30 [reviewed in 68]. Initially SAP18 was identified by yeast two-hybrid analysis, GST pull-down and co-immunoprecipitation assays as an INI1 and IN interacting protein [52]. Subsequently, several different members of the Sin3a-HDAC1 complex were specifically detected in HIV-1 virions including Sin3a, Sap18, Sap30, and HDAC1 [52]. Sorin *et al.* reported that they are incorporated in an IN dependent manner which likely involving a protein complex including INI1. The significance of HDAC1 activity in virions was tested by overexpression of a dominant negative HDAC1 mutant (H141A), by reducing HDAC1 expression using siRNA, or using chemical inhibitors of HDAC activity [52]. These approaches resulted in reduced (∼3-fold) HIV-1 infectivity suggesting that HDAC activity was important for early steps in HIV-1 infection. This was not attributed to a defect in virus maturation or ability of HIV-1 to fuse to a target cell. Curiously, a substantial defect in synthesis of reverse transcription products was observed, 10 to 100 fold, which indicated that a step between uncoating and reverse transcription was affected by HDAC1. Overall, the decreased infectivity was somewhat modest compared to the observed defect in viral cDNA synthesis. Nevertheless, components of the Sin3a complex, which includes INI1, plays a role in early steps of HIV-1 replication.

Exploration of the role of Sin3a, SAP18, Sap30 and HDAC1 should provide significant insight to virus replication strategies. Sin3a, as previously mentioned, is a scaffold protein, but no specific functions are known for Sap18 and Sap30 other than they are associated within stable protein complexes often involving transcription factors. It is possible that they perform a similar function by stabilizing the reverse transcription or pre-integration complexes. While HDAC1 activity is important for HIV-1 infectivity, the viral substrate for HDAC1 is unknown. The only HIV-1 proteins regulated by HDAC and histone acetyltransferase activity (HAT) are Tat and IN [69–74]. Acetylation of K51 in Tat positively affects HIV-1 gene expression [69,71,75,76], and this post-translational modification can be opposed by human sirtuin 1 (SIRT1) [77], a nicotinamide adenine dinucleotide-dependent class III protein deacetylase. Integrase can be acetylated by p300 on three lysine residues, K264, K266, and K273 in the C-terminal domain. Interestingly, mutating IN residues K264 or K266 resulted in defective reverse transcription [47] opening up the possibility of regulation of IN function by a reversible post-translational modification by p300 and a deactylase activity.

### 5.3. Survival motor neuron complex member Gemin2; an essential reverse transcription co-factor

Gemin2 (also called SMN-interacting protein 1, SIP1) is 280 amino acid, 30 kDa protein and a component of the survival motor neuron (SMN) complex composed of SMN, Gemins2–8 and Unrip proteins [reviewed in 78]. The primary role of the SMN complex in the cell is in the biogenesis of small nuclear ribonucleoproteins, which are assembled in the cell cytoplasm and accumulate in specific structures in the nucleus called gems (Gemini or coiled bodies) [79]. Gemin2 was originally identified as an IN binding protein in a yeast two-hybrid protein interaction assay [55], where Gemin2 amino acids 137 to 238 mediate binding to the C-terminal domain (amino acids 213–288) of HIV-1 IN (Table 1)(Figure 3). Hamamoto *et al.* showed that downregulation of Gemin2 expression by siRNA in primary monocyte derived macrophage cells significantly reduced HIV-1 replication that was attributed to reduced efficiency of reverse transcription and integration [55]. Thus, Gemin2 is required for an early event post-infection that affects early reverse transcription product (negative strand strong stop) less than later products or integrated DNA, suggesting that Gemin2 associates with either the reverse transcription or pre-integration complexes [55]. Alternatively, Gemin2 could recruit other cellular factors required for reverse transcription. For example DHX9 (RNA helicase A) is putative cellular factor regulating reverse transcription [80] that can associate with the SMN complex [81]. It is possible that other SMN complex proteins are also important. The difficulty in resolving this issue is that the steady state level of Gemin2 and SMN appear to be co-dependent. That is, the downregulation of either protein by siRNAi leads to reduced levels of the other [55]. Thus determining a role for SMN in early replication that is distinct from Gemin2 is problematic. Curiously, only one of four recent genome wide screens using RNAi methods [82–85] identified Gemin2 [82] as a HIV-1 human dependency factor, and SMN was not identified in any screen. The precise mechanism of how Gemin2 affects early replication remains to be determined.

## 6. Do Cellular Factors Directly Affect Reverse Transcription via RT Association?

The importance of host cell factors for HIV replication was recently highlighted by siRNA library genomic screens [82–85]. Each screen identified approximately 200–300 genes which affected HIV replication, with minimal overlap of genes between the individual screens. Interestingly, three of the studies identified host factors important for reverse transcription [83–85]. As there are other recent analyses of these genomic screens [86–88], in the section below we will instead focus on specific examples in the literature in which there is evidence of RT associations that affect reverse transcription.

### 6.1. Human antigen R (HuR): an effector of multiple steps of HIV-1 replication

HuR (also referred to as ELAVL1, HuA and MelG), is a ubiquitously expressed 326 amino acid, 36 kDa nuclear protein that possesses nucleocytoplasmic shuttling capabilities [89]. Like all members of the ELAV-like protein family, HuR exhibits high specificity and affinity for AU-rich elements (AREs) and contains 3 RNA binding domains. A direct correlation between HuR expression and mRNA decay has been demonstrated, thus implicating a role in the ARE-mediated mRNA degradation pathway [90–93]. A protective role for HuR in this pathway was suggested by the observation that overexpression of HuR in mouse cell lines significantly increased the stability of both type I and type II ARE-containing mRNAs [89,94]. Interestingly, studies have suggested that nucleocytoplasmic shuttling of HuR may regulate its function and whilst the precise mechanism behind the nuclear export of the protein remains unclear, it is most likely that HuR binds ARE-containing mRNAs in the nucleus, accompanies them to the cytoplasm, and then returns to the nucleus upon release.

Recently, altering HuR levels in cells has been shown to affect early HIV-1 replication [95] (Table 1)(Figure 3). Both depletion and overexpression of HuR verified the requirement of this protein for optimal reverse transcription. While the mechanism behind this activity remains unclear, it appears to be due to HuR an interaction with the RNase H domain of RT [95], as opposed to the previously discussed protein-RNA binding that regulates the role of HuR in mRNA stability. It is also possible that such activities are dependent on the presence of cellular factors that bind and regulate HuR. For example, HuR is incorporated in cellular stress granules that are common sites of regulation of mRNA stability and translation [96–98]. Interestingly human APOBEC3G (hA3G), known to have negative effects on reverse transcription [99–102], also localizes to stress granules [103]. Furthermore, hA3G was found to associate with a number of ribonucleoproteins present in stress granules [103], a significant proportion of which are known mRNA-interacting proteins including DHX9 [80,104], HNRNPU [105], PABPC1 [106], YB-1 [83,107] and SNRPA [83] that have previously been shown to affect various steps of HIV-1 replication including reverse transcription. Most significantly, a direct interaction between hA3G and HuR was revealed [103]. Hence, a HuR/hA3G protein complex could regulate the RTC as it progresses through the cytoplasm. Alternatively, HuR or DHX9 could oppose the negative effects of hA3G on HIV-1 reverse transcription. Further analysis of will be required to clarify a role for HuR:RT interaction.

### 6.2. A kinase anchor protein 1 (AKAP1): anchoring HIV-1 reverse transcription?

AKAP1, more commonly referred to as AKAP149 or PRKA1, is a member of the AKAP family of proteins which bind the regulatory subunits of cAMP-dependent protein kinase A (PKA) and anchors them to various membranes throughout the cell [108,109]. AKAP149 binds both RI and RII subunits of PKA, attaching them to the outer mitochondrial membrane [110], where it also interacts with c-myc binding protein (MYCBP, also called AMY-1), phosphodiesterase 4 (PDE4) and potentially caveolin 1 (CAV1) [111–113]. In addition to mitochondria, AKAP149 also exhibits localization to the endoplasmic reticulum and nuclear envelope where it assists the maintenance of nuclear integrity through interactions with B-type lamin (LMNB) and protein phosphatase 1 (PP1) [114–117]. A feature of the AKAP149 protein is the presence of a KH-Tudor domain [108] that facilitates both RNA-binding and self-association properties [118,119].

Recent research has demonstrated a direct interaction between AKAP149 and HIV-1 RT [95] (Table 1). Like HuR, AKAP149 binds to the RNase H region of RT [124]. While siRNA knockdown experiments indicate that this interaction is essential for optimal reverse transcription, a direct role for AKAP149 in reverse transcription requires confirmation. AKAP149 is not incorporated into virions and localizes to the endoplasmic reticulum, nuclear envelope and mitochondria [114,117,120]. While point mutation of the RNase H domain sharply downregulate AKAP149:RT interaction and reverse transcription, a clear connection has not been established. How AKAP149 is able to interact with RT prior to its function in reverse transcription is currently unknown, however AKAP149 does interact with cellular factors that could affect reverse transcription. For example, AKAP149 anchors PKA and PDE4 to mitochondria, both of which have been implicated in the regulation of HIV-1 replication [121,122]. PKA is incorporated into HIV-1 virions and can phosphorylate HIV-1 CA, an activity that may be important for viral infectivity [123]. Also, PKA and PDE4 themselves are key players in the cAMP signaling pathways [124], which can stimulate HIV-1 from latently infected cells [122,125] and in turn, can be activated by HIV-1 in normal lymphocytes [126]. Hence AKAP149 could affect reverse transcription indirectly, perhaps through a protein kinase dependent signaling pathway.

### 6.3. DNA topoisomerase 1 (TOP1) and increased RT efficiency

TOP1 is a 91 kDa sumoylated enzyme that regulates the topologic state of DNA during the process of transcription. TOP1 activity is required for optimal replication of many different DNA and RNA viruses including equine infectious anaemia virus (EIAV), Rous sarcoma virus (RSV), herpes simplex virus 1 (HSV1), adenovirus type 5 (Ad5), simian virus 40 (SV40) and HIV-1 [127–132].

TOP1 has been shown to enhance HIV-1 cDNA synthesis [133,134], thus implicating a role for this enzyme in reverse transcription (Table 1)(Figure 3). Interestingly, TOP1 has been shown to directly interact with HIV-1 NC [134], which itself is essential for the initiation of reverse transcription [135]. Additionally, *in vitro* reverse transcription assays have demonstrated that TOP1 can significantly enhance the activity of HIV-1 RT [134] and this can be impeded by the addition of the TOP1 inhibitor camptothecin [134]. While the exact mechanism behind this activity has yet to be elucidated, TOP1 may dissociate RT from structured RNA [136] in an ATP-dependent manner [137,138]. Alternatively, an enzymatic difference between cellular and virus-derived TOP1 has been suggested although this notion is controversial [131,134,137].

Also interesting is the ability of TOP1 to regulate virus tropism. HIV-1 virions derived from African green monkey cells are known to be less infectious than those derived from human cells [139]. Studies by Shoya *et al* [133] demonstrated that expression of human TOP1 in African green monkey cells increases the infectivity of progeny virions to 50–60% of that observed from human cells which was attributed to enhanced reverse transcription. Further research is required to determine the mechanism by which TOP1 enhances reverse transcription process. This could reveal whether this TOP1 activity is a possible candidate for the development of antiretroviral therapy.

## 7. Conclusions

Given that HIV-1 encodes only 9 genes and 15 proteins, it is not surprising that HIV-1 uses many hundreds of cellular factors during the virus replication cycle. Recent reports of human genome screens using RNAi methods and proteomic analysis have identified a plethora of human dependency factors, but few have been studied in detail. The question of which cellular components are required for productive reverse transcription will undoubtedly have a complicated answer. The RT:IN complex recruits cellular proteins that are required to complete reverse transcription, and its highly likely that other viral factors, such as NC, Vpr, MA, and Tat do the same. The research highlighted here begins to reveal how some factors regulate RT activity, but whether these factors are RTC components, or if they direct the RTC is unknown. The proteins discussed here provide a starting point towards understanding complex host-pathogen interactions that makes productive reverse transcription in cells possible. The investigations of cellular factors give hope that unraveling the critical interactions will ultimately be the key to novel treatments for HIV-1 infection.

## Figures and Tables

**Table 1.** Summary of host factors that affect reverse transcription.

**Host factor**	**Found in virus particle**	**Virus binding partner**	**RT/IN binding site**	**Affect on reverse transcription**	**Other binding partners with respect to RT**	**Reference**
**INI1**	Yes	IN	NH_2_-terminal; zinc-finger region	Unclear, recruits Sin3a complex proteins to the virion	HIV-1 TAT; SAP18	[52,53,61,63,66]
**SAP18**	Yes	IN	n/d	unknown, recruits Sin3a complex proteins to the virion	INI1	[52]
**HDAC1**	Yes	nil	n/a	required for early reverse transcription	INI1	[52]
**Gemin2**	No	IN	C-terminal region; amino acids 213–288	required for early reverse transcription	SMN1; DHX9 (but indirectly through the SMN complex)	[55,80]
**HuR**	n/d	RT	RNAse H domain	required for early reverse transcription	APOBEC3G	[95,103]
**AKAP149**	No	RT	RNAse H domain	required for early reverse transcription	PKA, PDE4,	[120,121,126]
**TOP1**	Yes	NC	n/a	stimulates RT activity	n/a	[132,134,135]
**APOBEC3G**	Yes, if Vif is absent	NC; viral RNA/DNA	n/a	inhibits early reverse transcription & inhibits elongation of viral cDNA	HuR	[99–102]
**DHX9 (RHA, RNA helicase A)**	Yes	Gag, viral RNA	n/a	required for early reverse transcription	SMN complex, APOBEC3G	[80,103]

n/d = not determined

n/a = not applicable
